# Examining the Differences in Format and Characteristics of Zoonotic Virus Surveillance Data on State Agency Websites

**DOI:** 10.2196/jmir.2487

**Published:** 2013-04-29

**Authors:** Matthew Scotch, Brittany Baarson, Rachel Beard, Robert Lauder, Aarthi Varman, Rolf U Halden

**Affiliations:** ^1^Center for Environmental SecurityBiodesign Institute and Security and Defense Systems InitiativeArizona State UniversityTempe, AZUnited States; ^2^Department of Biomedical InformaticsArizona State UniversityScottsdale, AZUnited States

**Keywords:** public health, zoonoses, World Wide Web, epidemiology, data analysis

## Abstract

**Background:**

Zoonotic viruses are infectious organisms transmittable between animals and humans. Agencies of public health, agriculture, and wildlife conduct surveillance of zoonotic viruses and often report data on their websites. However, the format and characteristics of these data are not known.

**Objective:**

To describe and compare the format and characteristics of statistics of zoonotic viruses on state public health, agriculture, and wildlife agency websites.

**Methods:**

For each state, we considered the websites of that state’s public health, agriculture, and wildlife agency. For each website, we noted the presence of any statistics for zoonotic viruses from 2000-2012. We analyzed the data using numerous categories including type of statistic, temporal and geographic level of detail, and format. We prioritized our analysis within each category based on assumptions of individuals’ preferences for extracting and analyzing data from websites. Thus, if two types of data (such as city and state-level) were present for a given virus in a given year, we counted the one with higher priority (city). External links from agency sites to other websites were not considered.

**Results:**

From 2000-2012, state health departments had the most extensive virus data, followed by agriculture, and then wildlife. We focused on the seven viruses that were common across the three agencies. These included rabies, West Nile virus, eastern equine encephalitis, St. Louis encephalitis, western equine encephalitis, influenza, and dengue fever. Simple numerical totals were most often used to report the data (89% for public health, 81% for agriculture, and 82% for wildlife), and proportions were not different (chi-square *P*=.15). Public health data were most often presented yearly (66%), while agriculture and wildlife agencies often described cases as they occurred (Fisher’s Exact test *P*<.001). Regarding format, public health agencies had more downloadable PDF files (68%), while agriculture (61%) and wildlife agencies (46%) presented data directly in the text of the HTML webpage (Fisher’s Exact test *P*<.001). Demographics and other information including age, gender, and host were limited. Finally, a Fisher’s Exact test showed no association between geography data and agency type (*P*=.08). However, it was noted that agriculture department data was often at the county level (63%), while public health was mixed between county (38%) and state (35%).

**Conclusions:**

This study focused on the format and characteristics of statistics of zoonotic viruses on websites of state public health, wildlife, and agriculture agencies in the context of population health surveillance. Data on zoonotic viruses varied across agencies presenting challenges for researchers needing to integrate animal and human data from different websites.

## Background

Data that are freely available on health agency websites can be used for surveillance, including monitoring infection rates over time and identifying outbreaks. This includes data on zoonotic viruses that are transmittable between animals and humans [1]. These viruses represent a significant population health concern [2], and their control is vital for reducing human and animal morbidity and mortality. In the United States, surveillance is done at the local, state, and federal levels [3]. Every state maintains departments of public health, agriculture, and wildlife. Often, these agencies rely on clinical and laboratory reporting of disease cases from predetermined lists [4] including those involving animals [5]. Depending on the state, reporting of animal cases can be separate from public health reporting [2]. A survey by Kahn [2] examined the reporting by practicing veterinarians to their state-appointed veterinarian who often work for agriculture agencies. Kahn found that 18% of state veterinarians responded that individual case reports of zoonotic viruses in animals are sent to public health agencies rather than to agriculture agencies [2]. As an explanation, Kahn notes the history of agriculture agencies was often to focus on agriculture and not necessarily surveillance of animal disease [2]. In a separate study by Kahn, a survey of over 1000 practicing veterinarians found that 30% notified their state agriculture agency in the event of an unusual infectious disease, while 23% notified their state public health agency [2]. On the public health side, a survey by M’ikanatha et al [6] asked state and local infectious disease epidemiologists what data sources they accessed for surveillance. Only 54% considered state agency websites their main resource of online investigation of public health data [6]. In another study, Staes et al [7] found that only 35% of clinicians during the 2009 influenza A H1N1 epidemic accessed state agency websites at least once a week while 50% never accessed them at all. In contrast, over half of the participants visited the CDC’s influenza website (53%) at least once a week [7]. These studies suggest inconsistent data reporting structures and utilization patterns across both human and animal health agencies.

One of the few Internet studies on zoonoses was a 2008 study by Pappas et al [8], which examined online resources for scientific information on zoonoses. The authors performed a Google and Yahoo! search for any content related to zoonoses or zoonotic infections [8]. The websites that were found included those sponsored by agencies at the international, country, state, and local levels [8]. Despite the global threat of zoonotic infections and the burdens that they have on developing countries, the authors found that the majority of the sites were from agencies or academic institutions within the United States [8]. Many of these sites were from state health agencies—a finding that is relevant to the scope of this paper and demonstrates their potential as a resource for zoonotic surveillance.

While there have been a limited number of studies focused on zoonoses, agency website data have been shown to be useful in other areas of health-related research and surveillance. A 2011 paper by Aswani et al examined state differences in reporting of central line-associated bloodstream infections (CLABSIs) [9]. The authors utilized data from agency websites for hospital acquired infection and CLABSI reports [9]. Searches from state websites included terms such as “health care quality, data, and/or statistical reports” ([9] pg. 388) or more specific terms related to CLABSIs or hospital acquired infection. Overall, the authors found variation in the data across the 15 states that had publicly available data; 86% of websites published data using infection rates while others did not provide a rate [9]. In addition, certain states adjusted their data by standardized infection ratios while others used device utilization ratios [9]. There were also differences in how the data were aggregated as some were done without consideration of the unit within the hospital [9]. The wide variation of data on agency websites makes state comparisons difficult and highlights the need for better standards in reporting.

At the federal level, the ArboNET system [10] is a collaborative effort to compile data on certain infectious diseases, most notably West Nile virus (WNV). It has been used in many surveillance research studies (such as [11-13]). It also includes data on St. Louis encephalitis, eastern equine encephalitis, La Crosse encephalitis, Powassan virus, and dengue fever. A study by O’Leary et al [12] used ArboNET to examine the severity of WNV post 1999. Cases between January 1, 2002, and March 15, 2003, were included in the study. The authors analyzed information such as date of illness and county of residence [12]. In total, over 4000 cases were included with 54% classified as *confirmed* and 46% as *probable* [12]; 84% of cases were from 11 states with the most cases from Michigan, Illinois, and Ohio [12]. Mississippi had the highest incidence when factoring in population size [12].

Federal surveillance initiatives such as ArboNET and the CDC’s influenza program [14] exist for some viruses; however, there are often many limitations with the data. Agencies follow their own procedures in terms of reporting [15] requiring careful consideration before aggregation with other states. There is often a loss of granularity as data are reported from states to the federal level. For example, CDC’s influenza surveillance program aggregates statistics by Health and Human Services (HHS)-defined geographic regions. This eliminates the ability to compare influenza statistics by state. In addition, ArboNet provides tables showing cumulative data by county per year, rather than providing monthly summaries. This eliminates the ability to compare statistics by month. They do provide more detailed data in the form of graphs; however, exact numbers can be difficult to interpret. Finally, important data such as gender and age of individuals might get removed as it is reported to the federal level prohibiting it from being used as a dimension for comparison. This eliminates the ability to compare differences by sex, race, and age groups. As data get aggregated, it becomes more and more difficult to uncover temporal, geographic, and demographic relationships of viral infection.

As an alternative, state agency data has the potential to be a valuable resource for epidemiologists and clinicians looking to analyze zoonotic viruses data among animals and humans. Current popular resources like ArboNET and HealthMap [16-18] have generated immense interest in the surveillance community and shown that integration of animal and human data can be of great value for monitoring of zoonotic viruses. However, less is known about the format and characteristics of state agency website data, despite the large amount of data that are collected. The purpose of this study is to examine websites of state agencies of public health, agriculture, and wildlife to characterize a subset of publicly available data for surveillance of zoonotic viruses. We explored issues beyond the mere presence or absence of data for a given virus and considered the quality, format, and completeness of the data for downloading and subsequent utilization for research purposes, including integration of human and animal data.

## Methods

We generated a list of 63 zoonotic viruses (Multimedia Appendix 1) from a review of Krauss [1]. We decided a priori, not to limit our focus to viruses that were endemic/enzootic to the United States, enabling rare events to be considered in our study. Owing to resource limitations and a desire to focus on the most recent data, the study was limited to the years 2000-2012 and to zoonotic viruses, excluding bacteria, fungi, and parasites.

For each of the 50 states, our search criteria consisted of the following steps:

1. Search on Google for *[state name]+ public health department* or *[state name] + wildlife department* or *[state name] + department of agriculture*. Two of the authors (RL and AV) examined public health websites, and two examined agriculture and wildlife websites relating to animal health (BB and RB).
2. Identify the appropriate website link from the Google search results.
3. Examine homepage for links related to surveillance data such as *epidemiological data*, *disease statistics*, *zoonotic diseases*, *animal health*, etc.
4. Use the website’s search function (if available) to find any data that might have been missed through navigation of the links.
5. Collect the following data related to a virus on the a priori list: the type of the statistic (including totals and averages), the years data are available (from 2000-2012), the infected host, and whether the data are in an HTML table, a free-text paragraph, or downloadable as a PDF file, Word document, or spreadsheet. We also considered the frequency of the data, the level of geographic detail, and the presence of any demographic-related data.
6. Summarize results for each category in #5 (except for *demographics*), prioritizing based on our estimation of the following: If researchers need to extract data from a website for analysis purposes, what might be their preferences of data format, level of geography, how it was presented, etc. For *geography*, the highest level of granularity is likely preferred for research compared to lower levels such as state totals. Thus, we chose *city* as the best scenario for the *geography* category. For this analysis, if virus data were provided at different levels for the same year, such as city and state, we considered it *city*. A *mixed* result was only indicated if for a given virus and year, it had one type of data (eg*,* as city-level data) and then for the same virus for a different year, had another type of data (eg*,* county-level data). This procedure was done in order to avoid binning everything into *mixed*.
7. Exclude from our results any links to external sites; thus, a website with a link to an outside source, such as another state agency or a federal agency, was not factored into our work.

Descriptive statistics and charts were done in Microsoft Excel. Hypothesis testing for comparison of proportions within public health, agriculture, and wildlife was done using SAS v.9.2. For this, we considered either the chi-square or Fisher’s Exact test.

## Results

**Figure 1:**
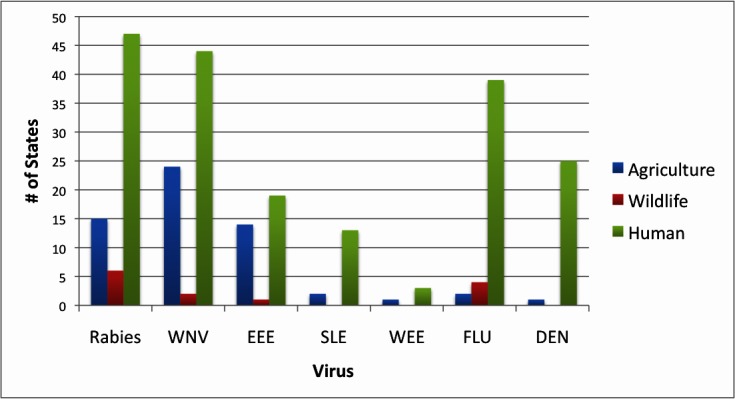
Common zoonotic viruses data provided by agencies of public health, agriculture, or wildlife on their websites.

All 50 states had websites for public health, wildlife, and agriculture agencies, although naming conventions did vary. From 2000-2012, state health departments, followed by agriculture, and then wildlife, most often had virus data. In total, seven viruses were common among all three groups. These included rabies, West Nile virus (WNV), eastern equine encephalitis (EEE), St. Louis encephalitis (SLE), western equine encephalitis (WEE), influenza, and dengue fever. Figure 1 shows the number of states with data for these viruses from 2000-2012. Rabies and WNV data were the most abundant. Rabies data were different from the other zoonotic viruses. Here, many public health agencies provided data on cases of infection in animals instead of agriculture or wildlife agencies. Since human cases of rabies in the United States are extremely rare, due to postexposure prophylaxis, there is often nothing to report. Thus, the 45 state public health agencies providing rabies data during this time span detailed animal cases and the rare exception of human cases. Concurrently, some wildlife or agriculture agencies did provide some rabies data, and thus there is a possibility of an overlap of rabies data. However, this occurred infrequently.

**Figure 2:**
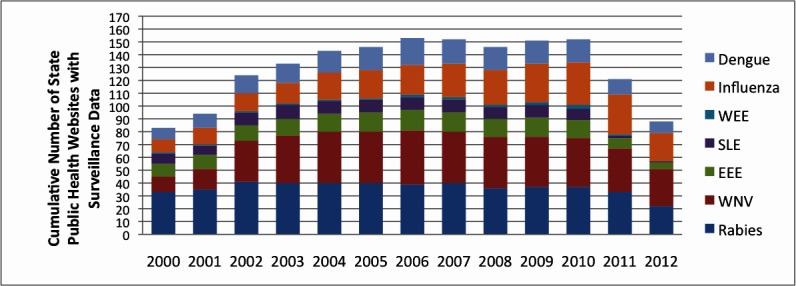
Cumulative number of state public health websites with surveillance data, by virus and year.

Figure 2 shows a temporal analysis of public health website data for all 50 US states, considering the seven common viruses (Figure 1). As of this writing, the year 2012 is not complete and since some websites might wait until the year is completed to provide statistics, this value is likely an underestimate.

Tables 1-3 compare by agency, the number of states that provided zoonotic viruses data for 2000-2012. If data were provided on a website for any year during 2000-2012, the value “1” was added to the column. The number of years was not a factor in this comparison, and thus 10 years of rabies data or 5 years still resulted in adding “1” to the column.

Table 1 considers the type of the statistic such as a total, rate, or percentage. We set *totals* as the priority for this category, believing that this statistic is the easiest to analyze across groups versus a rate or ratio where the at-risk population, especially for animals, is not usually known. Here, the majority of the website data of all three agency types were provided as totals. Thus rates, percentages, other types of statistics, and agencies that provided a mix of formats were grouped into the *other* group. We also considered the level of geographic granularity, as this impacts conclusions that can be drawn from a population-level analysis. As mentioned, *city* was set as the priority with the remaining order based on granularity (ie, county then state, etc). A Fisher’s Exact test showed no association between geography data and agency type (*P*=.08). However, public health agencies, which mostly provide data on human cases, had a proportion of 38% at the county level versus agriculture agencies that provided 63% of their data at the county level. This could be related to the concern over issues of confidentiality among small geographic sample sizes. The *mixed* variable represents an agency that provided virus data at different levels of granularity for different years, such as one year at the county level and one year at the state level. Finally, the analysis of demographics suggested that when data were provided, they usually did not include age, gender, or race of humans. Lack of data on demographics limits comparisons across datasets and the ability to identify the most at-risk groups. For example, if agriculture and wildlife agencies do not provide the infected host (eg, bats, skunks) when providing statistics on virus cases, comparisons across different host populations are limited.

We also considered the format of the data (Table 2). Here, we set *spreadsheet* such as XLS or CSV files as the priority since these files can easily be downloaded and formatted and are already in an application that supports analysis and comparison. HTML was second since content on a webpage can often be copied and pasted as needed. The format of the data impacts the feasibility of data integration. For public health agencies, 68% of the data was provided through links to PDF documents, which often requires manual data entry into a researcher’s database or spreadsheet. A higher percentage of agriculture website data was available in HTML format than public health (61% versus 19%). Finally, we also analyzed the manner in which the data were presented. Tabular data (the priority) is potentially easier to load into a database or spreadsheet, while graphs and charts are visually informative yet sometimes the actual numbers are not provided, thereby forcing the researcher to infer the true numbers. Public health departments had a much greater percentage of data in tables (57%), while agriculture agencies mostly provided their virus statistics within text (63%). This presents challenges for researchers relying on automated updates (data dumps) or “Web crawlers” to perform Natural Language Processing (NLP) on the free-text in order to extract the statistics.

Table 3 considers the frequency in which data are provided as well as the number of years that the data are available. Public health departments most often provided data as annual aggregates (66%) as opposed to weekly or monthly trends. Conversely, agriculture or wildlife agencies more often provided data *as identified* (58%) and typically in a paragraph of free-text such as describing a recent case of WNV in a red-tailed hawk.

We also considered the time-span of reporting since gaps over time limit the ability for researchers to identify true temporal fluctuations in the data. An agency might provide data for cases from 2000-2005, but for a variety of reasons (loss of Webmaster or epidemiologist, perceived lack of interest in the community, etc) not include the data after that period. Here, the average number of years that data for a given virus were available on their website was greater among public health departments than departments of agriculture and wildlife. This is likely associated with the higher proportion of *as identified* data among the animal agencies (agriculture and wildlife).

For public health agencies, it was interesting that highly prevalent viruses such as influenza had the smallest number of years available (7.1). This might be related to the fact that the CDC and their surveillance program [14] offer historical data on a weekly basis, and thus many agency websites contain a link to the CDC for this information. Since we did not consider external links in this study, this was not counted in our totals.

**Figure 3:**
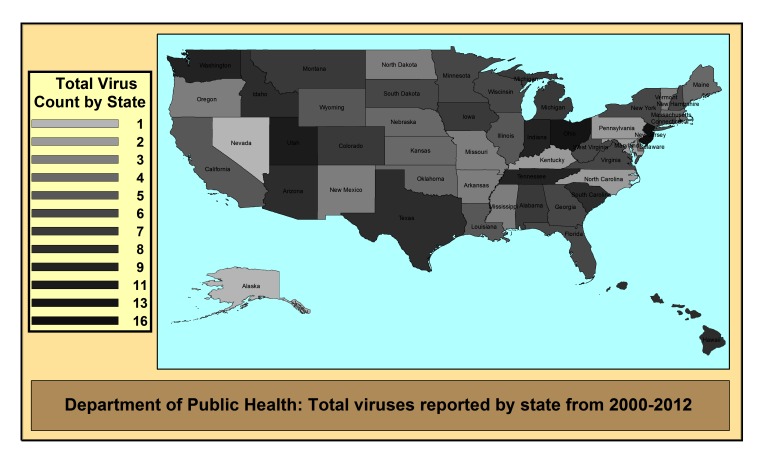
Map of the number of online virus data at public health departments (theoretical maximum: 63 viruses).

**Figure 4:**
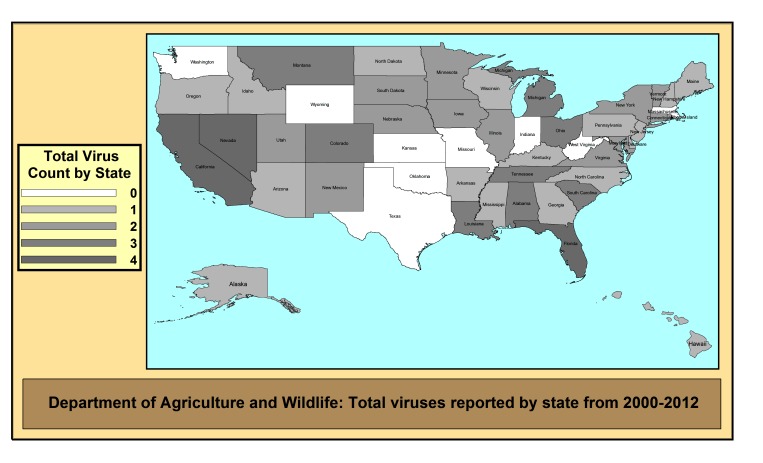
Map of the number of online virus data at agriculture and wildlife agencies.

We also mapped total virus data by the different agencies during 2000-2012 (Figures 3 and 4) and considered all 63 viruses from our list (Multimedia Appendix 1). The presence of the virus data even once during the time-span was counted towards the total (hence the number of years was not a factor). Thus, the theoretical maximum for each state was 63 since that was the size of our a priori list. Since many of the viruses on our list are not found in the United States, the numbers were much lower. In addition, we did not consider any links to external sites in our results, thus a state department of agriculture that links all of their data to the US Department of Agriculture (USDA) would seem to have less data in our results than a site that did not link out. Geographic influence appeared to occur in some areas of the country, as states in the middle had similar numbers, yet areas in the northeast such as Ohio and Pennsylvania or southwest such as Arizona and New Mexico were different from one another. The map of the two animal agencies (Figure 4) is greatly impacted by the number of links to external data sources. This is evident with a state like Texas, which did not provide any statistics on their sites, instead providing links to other sources such as the state public health agency or the USDA.

**Table 1:** Comparison by agency type of the number of states that provided zoonotic viruses data on websites by type of statistic used, level of geography, and inclusion of demographics, 2000-2012 (data combined for each of virus in Figure 1; *P* values determined by chi-square for Statistic and Fisher’s Exact test for Geography; percentages may not add to 100% due to rounding).

		Agencies			
		Public Health (N=187)	Agriculture (N=58)	Wildlife (N=11)	*P* value
**Statistic** ^a^					.15
	Totals	167 (89%)	47 (81%)	9 (82%)	
	Other^b^	20 (11%)	11 (19%)	2 (18%)	
**Geography**					.08
	City	13 (7%)	3 (5%)	0	
	County	72 (38%)	37 (63%)	4 (31%)	
	State	66 (35%)	13 (22%)	7 (54%)	
	Mixed	31 (16%)	5 (8%)	2 (15%)	
	Other^c^	5 (3%)	1 (2%)	0	
**Demographics** ^d^					
	Age	51	5	0	
	Gender	32	2	0	
	Race	13	0	0	
	Animal host	80	54	11	
	None	84	3	2	

^a^Additional *none* variable not shown. There were three instances where a website indicated that “no cases” were reported.

^b^Includes *rates*, *percentages*, *mixed*, or *other*.

^c^Includes *zipcodes*, *region*, *town*, *district*, *jurisdiction.* There was one instance of zipcode-level data.

^d^Percentages not used since categories are not mutually exclusive. Base “N” for each agency type will not add up since categories are not mutually exclusive. Removed *other,* which was zero for all agencies.

**Table 2:** Comparison by agency type of the number of states that provided zoonotic viruses data on websites by format and presentation, 2000-2012 (*P* values determined by Fisher’s Exact test; percentages might not add to 100% due to rounding).

		Agencies			
		Public Health	Agriculture	Wildlife	*P* value
**Format**					.001^a^
	Spreadsheet	3 (1%)	0	0	
	HTML	36 (19%)	36 (61%)	6 (46%)	
	PDF	128 (68%)	20 (33%)	5 (38%)	
	Mixed	20 (11%)	3 (5%)	2 (15%)	
**Presentation**					
	Table	108 (57%)	5 (8%)	2 (15%)	
	Graph	16 (9%)	0	1 (8%)	
	Map	6 (3%)	6 (10%)	0	
	Text	13 (7%)	37 (63%)	5 (38%)	
	Mixed	44 (23%)	11 (18%)	5 (38%)	

^a^Computed by removing *other* variable with all zeros.

**Table 3:** Comparison by agency type: The number of states that provided zoonotic viruses data on websites by frequency as well as the average duration of years the data is provided by virus, 2000-2012 (viruses chosen from Figure 1; frequency *P* value determined by Fisher’s Exact test and virus *P* value determined by ANOVA; percentages might not add to 100% due to rounding).

		Agencies			
		Public Health	Agriculture	Wildlife	*P* value
**Frequency**					<.001^a^
	Weekly	25 (13%)	3 (5%)	0	
	Monthly	8 (4%)	2 (3%)	0	
	Yearly	124 (66%)	18 (30%)	3 (23%)	
	Mixed	24 (13%)	2 (3%)	1 (8%)	
	As identified	6 (3%)	34 (58%)	9 (69%)	
**Virus (average # years)** ^b^					<.001
	Rabies	9.3	3.4	4.2	
	WNV	10.0	3.5	7	
	EEE	8.5	2.6	1	
	SLE	8.2	5	0	
	WEE	6	9	0	
	Influenza	7.1	1.5	2	
	Dengue	7.7	1	0	

^a^Computed by removing *other* variable with all zeros.

^b^Only includes states that reported data for a given virus.

## Discussion

Zoonotic viruses data varied across agencies of public health, agriculture, and wildlife, in relation to many of the different factors. Considering the amount of data available, wildlife agencies clearly had the least. This limits the ability to make informed decisions regarding the impact of zoonotic viruses among wildlife populations. The larger amount of data for public health and agriculture agencies are likely due to the fact that they receive provider-oriented data from clinicians and laboratories. Meanwhile, wildlife agencies rely on mortality events reported by the public, as the case with dead crow sightings for WNV [2] or initiatives like hunter-harvest programs.

Pennsylvania’s WNV data [19] was one of the examples that we considered as having excellent data characteristics. It has specific dates and county-level data from 2001-2012. They also include demographic data for age and gender. While it does not have Excel format, the counts are in HTML tables with tables and maps (which can easily be copied into a spreadsheet). Finally, it had human, bird, mosquito, and veterinarian data enabling for easy comparison. Despite the larger amount of data for public health and agriculture agencies, differences exist that provide challenges for integration and analysis of zoonotic viruses. We now discuss the implications of our findings related to geography, format, presentation style, and frequency of the data.

### Geographic Mismatches Between Datasets

Results suggested that public health agencies provided fewer data at higher levels of geographic granularity than agricultural departments; however, no statistical difference was found (*P*=.08). As mentioned, this might be related to issues of sensitivity and confidentiality of data, especially in small geographic areas. When integrating disparate datasets for a given virus, a common practice is to use the highest matching level of granularity. Given that public health data are often provided at higher geographic levels, this will result in a loss of granularity for animal health data. One potential approach is to use data mining and classification algorithms, such as decision trees, to predict the data at a higher level of granularity; however, the accuracy of this technique for geographic inference is not known.

### Format and Presentation Styles

Of the public health data, 68% were provided in PDF file format. This impedes processing and analysis of the data, as manual database entry is often required. Web 2.0 and 3.0 technologies can partially address this problem including the use of software that can convert PDFs to text, thereby enabling easier modification. Even data in HTML format can be a challenge. Our findings showed that the majority of agriculture agencies had their data embedded in the webpage. For this, applications such as screen scrapers like Yahoo!’s Dapper [20] enable content to be extracted and automatically placed into an electronic format. Prior work by Yang et al [21] developed a framework for screen scraping climate data from websites, and work from Moumtzidou [22] considered environmental health data.

Agencies differed on their presentation style, with public health departments using more tables to present their data. Tables often lend themselves to easier extraction as they can be copied and pasted into a spreadsheet for processing. In addition, screen scrapers tend to work well with tables, although this can vary depending on the layout of the table. Conversely, agriculture and wildlife agencies presented their data within text of the webpage. This likely corresponded to their tendency to provide data *as identified* as a written description*.* This presents challenges for researchers as they are forced to scan the text and manually identify relevant data. As previously mentioned, one solution is to use Natural Language Processing (NLP) to automatically extract relevant information from the text. Much of the work in the NLP community has focused on extraction of health information from the social media (such as Twitter) as opposed to health content sites [23]. One example by Doing-Harris et al [24] uses NLP for an online consumer health website. Another focus of NLP has been on mining and characterization of public health reports. Examples include research groups employing EpiSPIDER [25] and Stewart et al [26] who performed text mining classification work on ProMed reports [27,28]. While promise has been shown in these areas, more work needs to be done by the NLP community for applying methods and approaches to extract surveillance data from health agency websites.

### Frequency and Gaps in Data

Our results indicate that temporal gaps do exist regardless of agency. While our decision not to consider external links may have resulted in a potential underestimation (see Limitations), gaps in providing data do exist and need to be considered when analyzing disparate data. Our findings showed that agriculture and wildlife agencies had very low averages for total years of data. As indicated, this might be related to their tendency to provide data *as needed*. However, the researcher must determine if these gaps are due to absence of cases or missing information. Consistency in reporting data on websites is important for accurate assessment of public health needs. Gaps or missing data due to inconsistent reporting can bias parameter estimates [29] and lead to underestimation of virus infection in the population. A solution is to compare state agency website data with other sources of information, including federal health sites, news reports (ProMed, HealthMap), and online public databases, such as the Food and Agriculture Organization’s (FAO) EMPRES-i [30], or electronic health record (EHR) and hospital utilization data. One resource for this is the Semantic Web, which was designed to promote interoperability of online resources and concepts, such as mash-ups facilitating integration of data across the Web [31]. Examples of public health applications that have used advanced and open-source Web technology include EpiVue [32] and SMCP-Aedes for dengue fever surveillance [33]. However, these systems do not address issues related to routine sharing and integration of animal and human health data.

### Recent Trends in Zoonotic Viruses Data on Agency Websites

Examining how characteristics in zoonotic viruses data changed over time across the three agencies will highlight trends for future surveillance efforts. For all of the categories measured in Tables 1-3, we graphed annual trends for each agency (Multimedia Appendix 2). For public health agencies, data in the year 2011 sharply decreased across all categories (in Multimedia Appendix 2, see Figures S1, S4, S7, S10, S13, S16). In Multimedia Appendix 2, Figure S1, the frequency of data was 145 in 2010, but only 123 in 2011. Similarly, geography of data was 150 in 2010 then declined to 122 in 2011 (Multimedia Appendix 2, Figure S4). Meanwhile, agriculture agencies had an increase across all categories for 2012, despite the year being incomplete. For example, the graph of frequency of data was 17 in 2011, while rising to 26 in 2012 (Multimedia Appendix 2, Figure S2). In addition, presentation of data was 17 in 2011, but 28 in 2012 (Multimedia Appendix 2, Figure S11).

There are many explanations for these findings. The sharp decrease in public health data for 2011 could be because more states might have increased their use of external links to data collected by the CDC rather than reporting it on their own websites. Since our study did not include these in our results, this would suggest a decrease in data. Another possibility is lack of time and resources dedicated to adding data to websites. Since many state public health budgets are small, there is a possibility that these departments have decided to allocate their time away from website reporting. Surprisingly, while public health agencies showed a decrease in 2011, agriculture agencies increased their data in 2012. There is a possibility that agriculture agencies have made more of an effort to post their data on their own websites rather than providing external links to CDC or USDA. These agencies might feel an obligation to keep the public informed as more people become aware of zoonotic viruses and the relationship between animal and human transmission.

Since one or two years does not provide enough evidence of a changing trend, additional work should focus on studying 2013 and beyond. Also, for zoonotic viruses, more research needs to focus on the potential of integrating animal and human data across state websites. Our study found seven common diseases in the United States. This provides for a great opportunity to utilize both animal and human sources for surveillance. In addition, for certain viruses, state-level detail might be used to augment more aggregated results at the federal level. Understanding how these different data sources can be utilized together might enable for more robust and elaborate surveillance systems.

Despite these challenges, agency website data offer great potential for virus surveillance by both clinicians and public health professionals. A survey by Gesteland found that 30% of clinicians in Utah access their states health department website [34]. However, the authors found that the main reason for not accessing the site was lack of awareness [34]. Thus informing clinicians about the potential benefits of public health data might increase utilization and provide valuable resources for clinical care.

### Limitations

The authors recognize several limitations with this work. First, we decided to prioritize variables (based on anticipated preference among researchers) within categories in order to reduce binning everything into a *mixed* category. Thus, lower priority variables such as *county* or *state* were omitted in the presence of higher priority variables such as *city*. Thus, our results underestimate the proportion of lower priority characteristics associated with surveillance data. However, our method was consistent across the three different agency types; thus, we feel that our results still provide informative findings into the characteristics of zoonotic viruses data among the different types of agencies.

Second, we did not consider external links as a data source in this study, such as state health department websites that send visitors to other agencies (such as the CDC or USDA). Thus, a state like Texas that does not provide animal health data directly on their site, received lower scores (Figure 4). This likely resulted in an underestimation of the amount of data available on these types of sites. In other variables, such as *frequency*, we likely overestimated the amount of gaps over time (if an agency decided to link to another source). However, the purpose of our study was to focus completely on the content in the website itself, and not as a data portal.

We considered a website as providing data if it was done at least once during our study period (2000-2012). We did not explore data that was received by agencies but not indicated on their website during the study period. Finally, due to resources and time constraints, we limited our work to zoonotic viruses. This implies that more work needs to be done to understand the availability and utility of health data relating to animal-borne bacteria, fungi, and parasites, as well as non-zoonotic viruses.

### Conclusions

This study focused on the format and characteristics of zoonotic virus statistics on websites of state public health, wildlife, and agriculture agencies in the context of secondary sources of surveillance and research data. Zoonotic viruses data varied across agencies presenting challenges for researchers needing to integrate animal and human data from different agency websites. Advanced Web technologies can partially address this, but more effort is needed from the biomedical informatics community to work with public health, agriculture, and wildlife agencies to address online data access, quality, and consistency in order to promote and facilitate integration of animal and human data for surveillance of zoonotic viruses.

Federal initiatives such as ArboNET and the CDC influenza program have limitations for granular-level data analysis including regional preparedness efforts. Geographic, temporal, and demographic information might be available through a state health department but become lost as it is aggregated for federal reporting. This makes it more difficult to uncover hidden drivers of viral infection in the population. Data on state websites has often been overshadowed by more popular federal initiatives but offer the potential to be a valuable and rich resource for zoonotic disease surveillance.

## Multimedia Appendix 1

List of 63 zoonotic viruses considered in this study.

## Multimedia Appendix 2

Temporal analysis by agency type.

## Abbreviations

ANOVA: analysis of variance
CDC: Centers for Disease Control and Prevention
CLABSI: central line–associated bloodstream infection
CSV: comma-separated values
EEE: Eastern Equine Encephalitis
EMPRES: Emergency Prevention System
FAO: Food and Agriculture Organization
HHS: Health and Human Services
HTML: HyperText Markup Language
NLP: Natural Language Processing
PDF: Portable Document Format
SLE: St. Louis encephalitis
USDA: United States Department of Agriculture
WEE: Western equine encephalitis
WNV: West Nile virus
