# Supplementary material for: Examining the Differences in Format and Characteristics of Zoonotic Virus Surveillance Data on State Agency Websites
Source: J Med Internet Res. 2013 Apr 29;15(4):e90. doi: 10.2196/jmir.2487 (PMC3650930; doi:10.2196/jmir.2487)
Supplement: Supplementary file 1 [file jmir_v15i4e90_app1.pdf]

Multimedia Appendix 1. List of 63 zoonotic viruses considered in this study.

Alphaviral Zoonoses
Bovine Spongiform Encephalopathy
Buffalopox
Camelpox
Chikungunya Fever
Colorado Tick Fever
Contagious Ecthyma of Sheep (Orf)
Cowpox
Creutzfeldt-Jakob Disease (new variant)
Crimean -Congo Virus hemorrhagic fever
Dengue Fever
Eastern Equine Encephalitis
Ebola Virus Hemorrhagic Fever
Elephantpox
Encephalopyocarditis
Epidemic Polyarthritis (Ross River Fever) & Barmah Forest Fever
Foot-and-mouth disease
Genus Coltivirus
Genus Orbivirus (Kemerovo Complex)
Hanta Virus Pulmonary syndrome
Hemorrhagic Fever with Renal syndrome
Herpes B Virus: Monkey Herpes Infection
Influenza Virus
Japanese Encephalitis
Kyananur Forest Disease and Alkhurma Virus Hemmorhagic Fever
La Crosse Virus
Lassa Fever
Louping III
Lymphocytic Chriomeningitis
Marburg Virus Hemorrhagic Fever
Mayaro Fever
Milker's Nodules (Pseudocowpox)
MonkeyPox
Murray Valley Encephalitis and Kunjin Virus Disease
Newcastle Disease
Nipah Virus Encephalitis
O'Nyong-Nyong Fever
Oropouche Virus
Papular Stomatitis
Powassan Virus Encephalitis
Rabies
Rift Valley fever
Rocio Encephalitis
sand fly fever
SARS
Semliki Forest Fever
Sindbis Fever
Snowshoe Hare Virus

St. Louis Encephalitis
Swine Vesicular Disease
Tahyna Virus
Tanapox Virus
Tick Bourne Encephalitis
Vaccinia Virus
Venezuelan Equine Encephalitis
Vesicular Stomatitis
Wesselsbron Fever
West Nile Fever
Western Equine Encephalitis
Yaba Monkey Tumor Virus
Yellow Fever
Zoonosis Caused by Hendra Virus
Zoonosis caused by New World Arenaviruses
