# Supplementary material for: Examining the Differences in Format and Characteristics of Zoonotic Virus Surveillance Data on State Agency Websites
Source: J Med Internet Res. 2013 Apr 29;15(4):e90. doi: 10.2196/jmir.2487 (PMC3650930; doi:10.2196/jmir.2487)
Supplement: Supplementary file 2 [file jmir_v15i4e90_app2.pdf]

Multimedia Appendix 2. Temporal analysis by agency type.

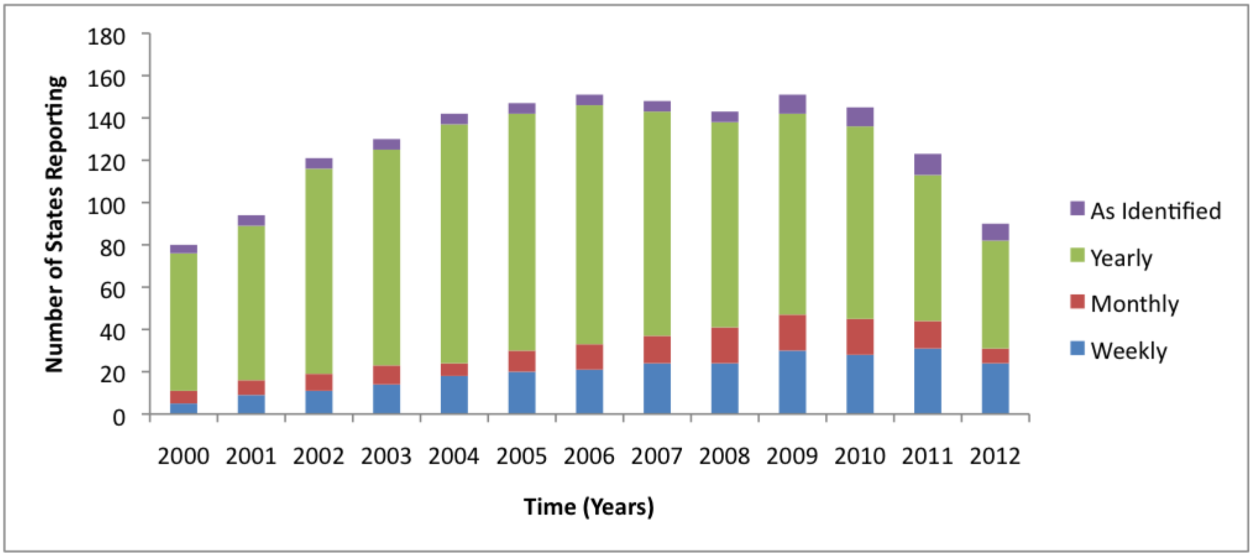

Figure S1: Public health agency frequency of data by year.

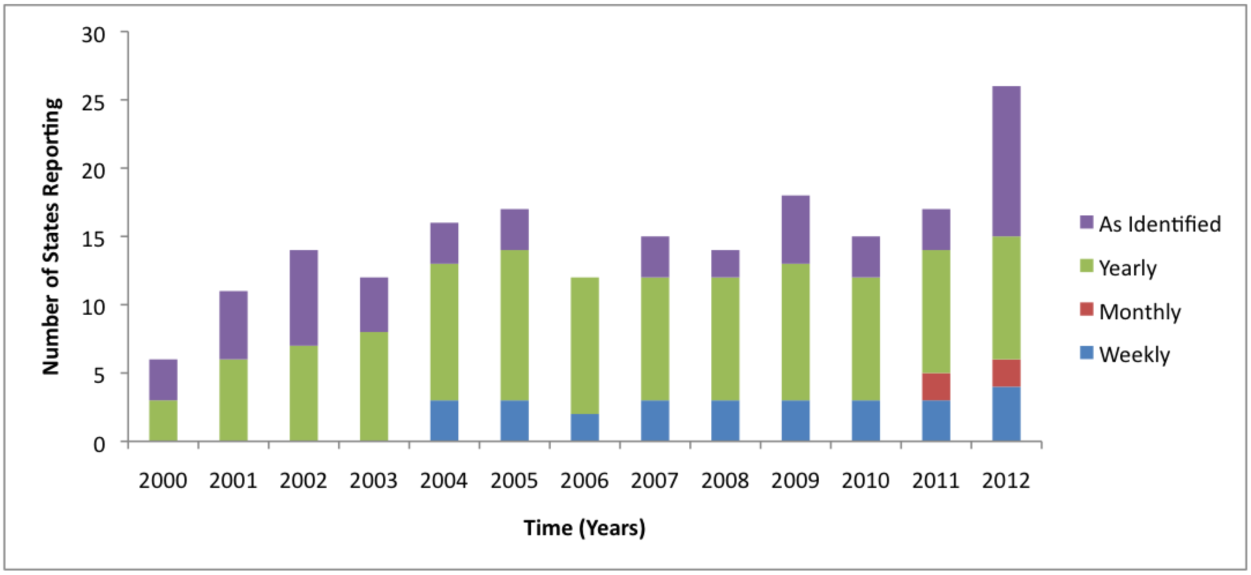

Figure S2: Agriculture agency frequency of data by year.

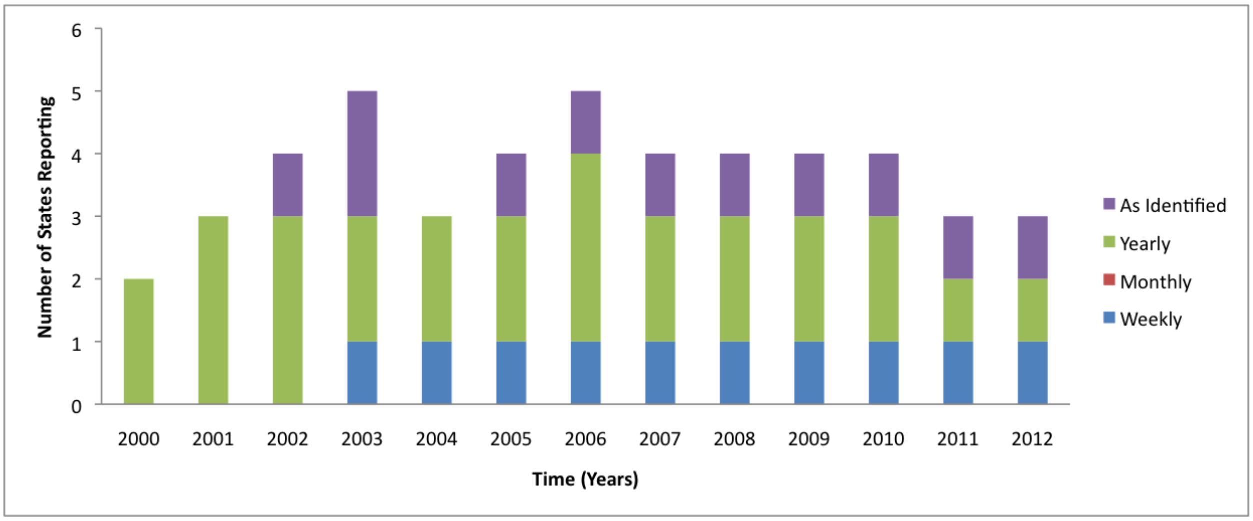

Figure S3: Wildlife agency frequency of data by year.

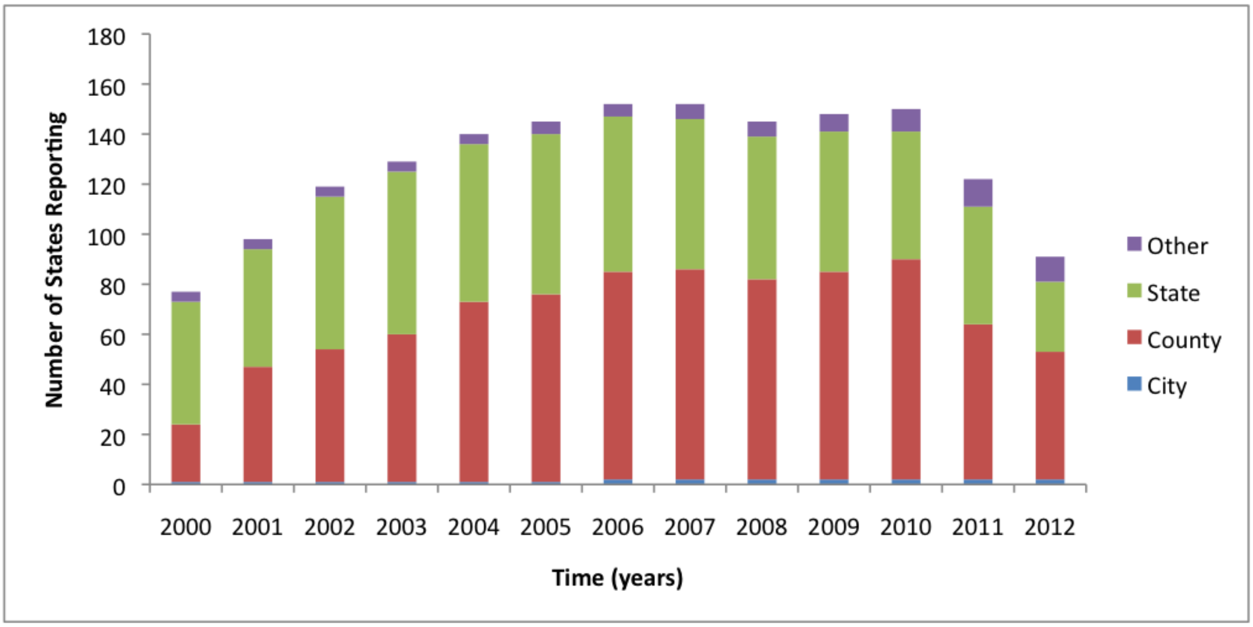

Figure S4: Public health agency geography of data by year.

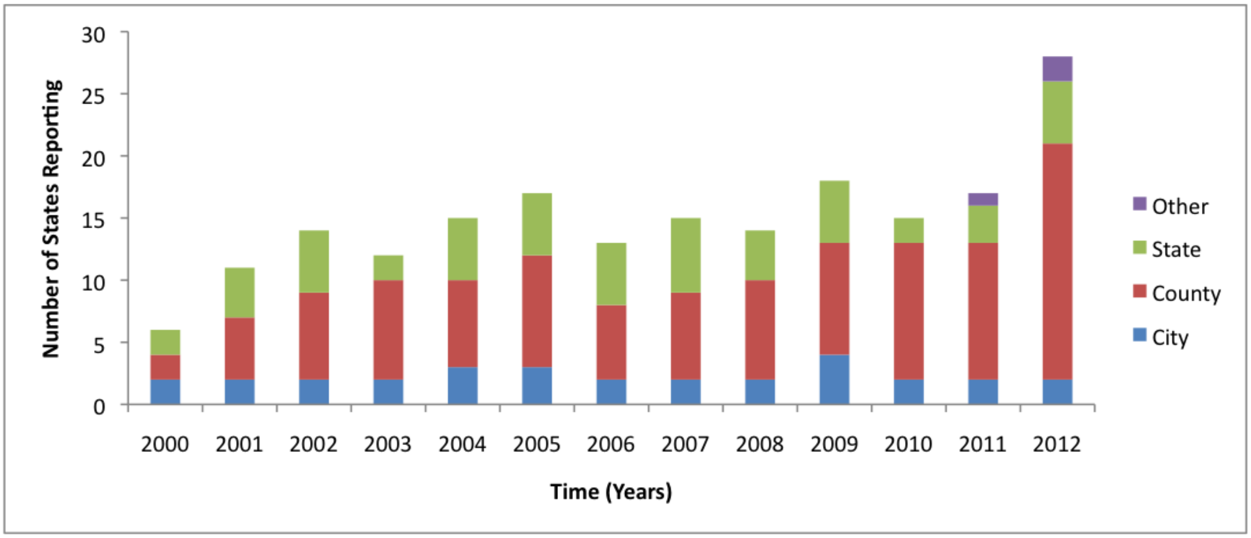

Figure S5: Agriculture agency geography of data by year.

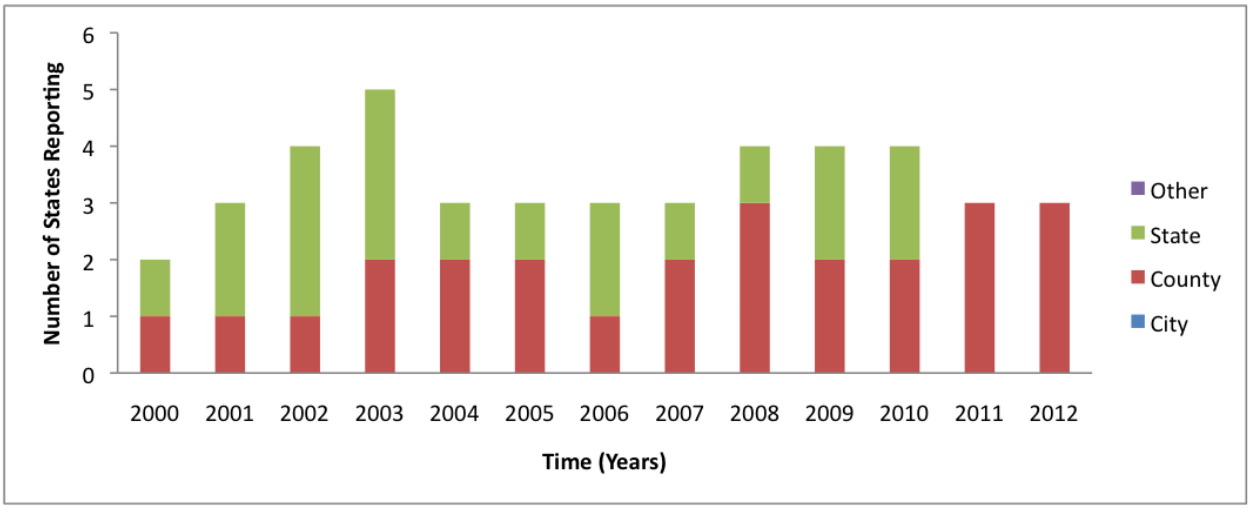

Figure S6: Wildlife agency geography of data by year.

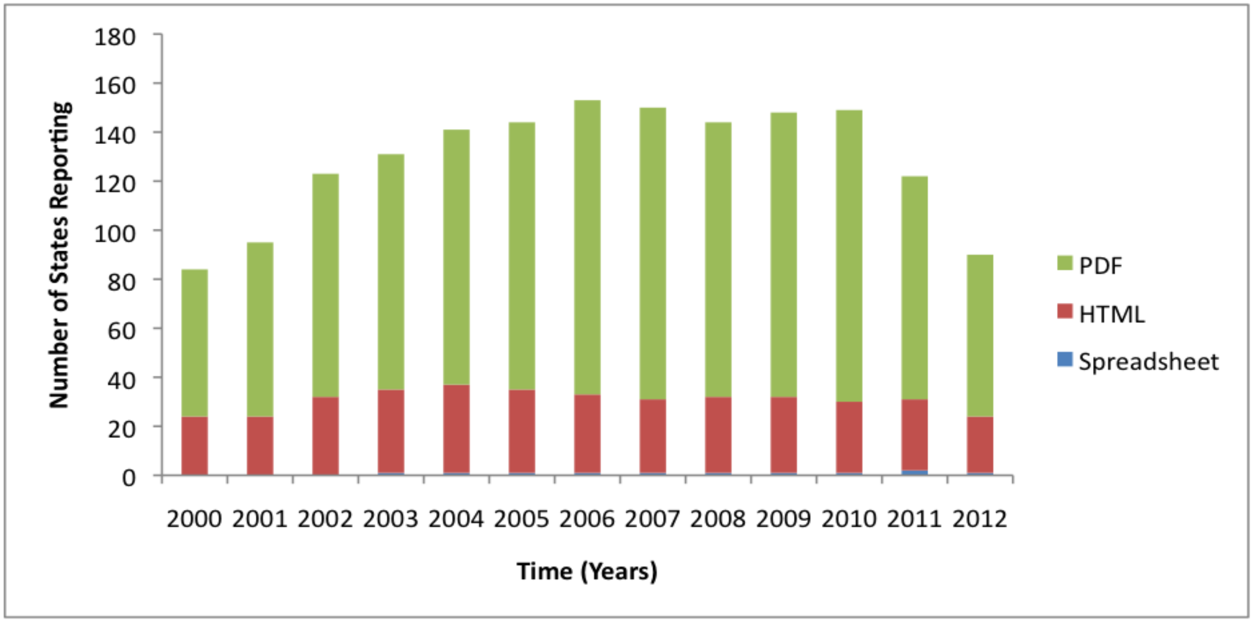

Figure S7: Public health agency format of data by year.

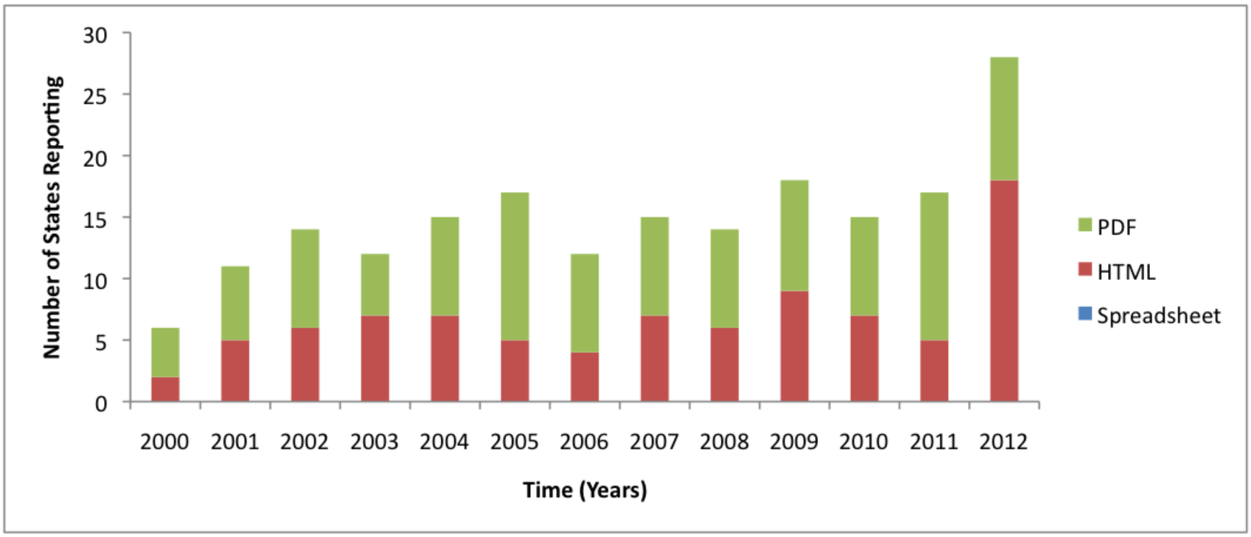

Figure S8: Agriculture format of data by year.

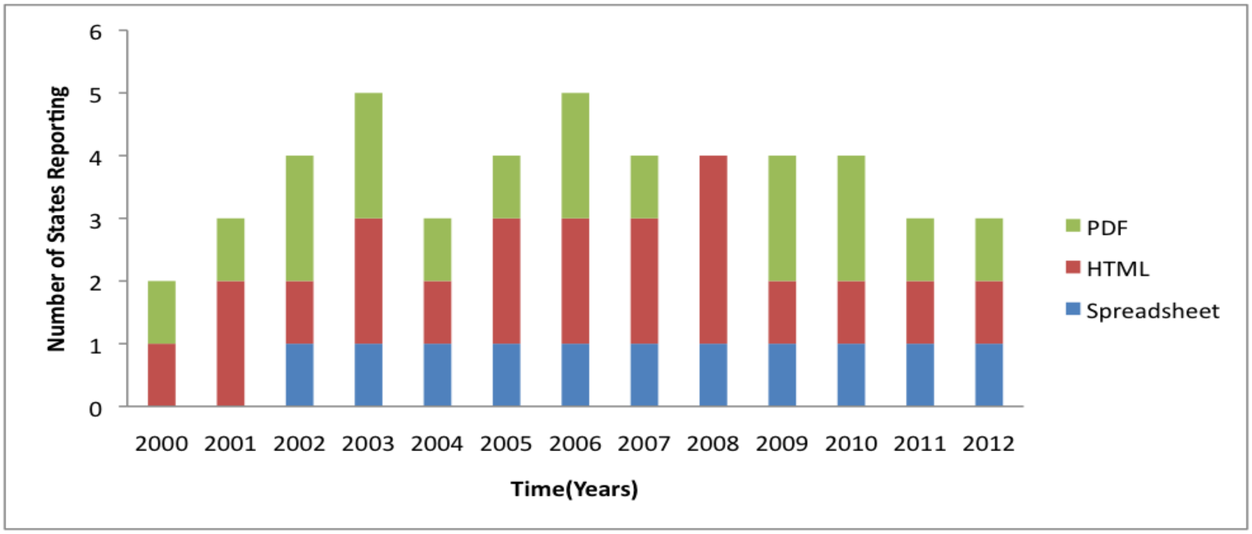

Figure S9: Wildlife agency format of data by year.

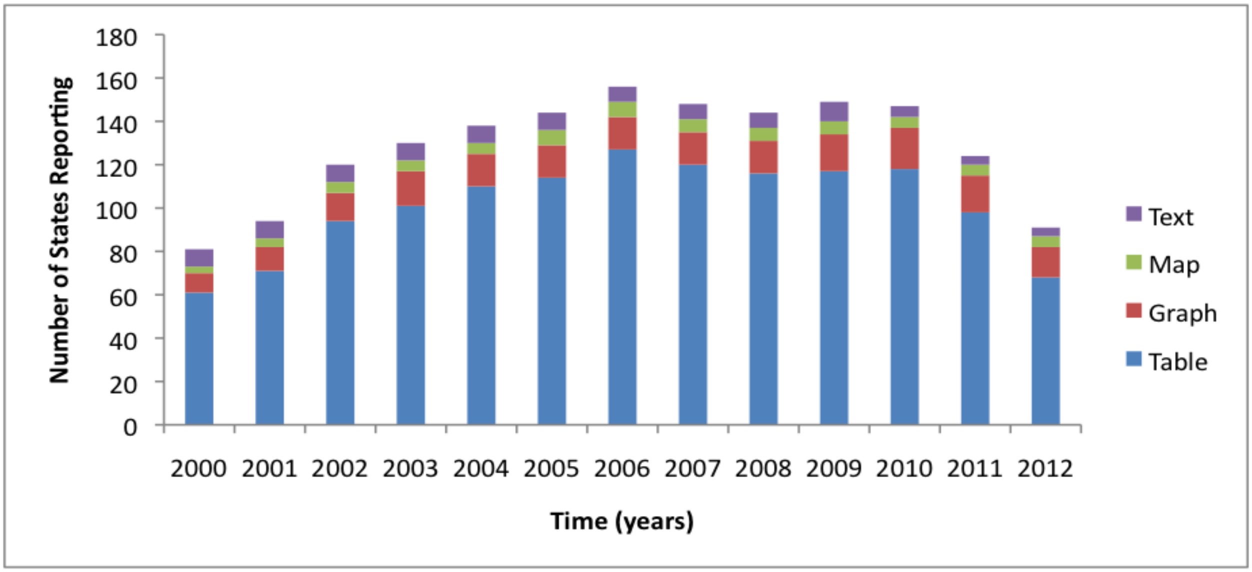

Figure S10: Public health agency presentation of data by year.

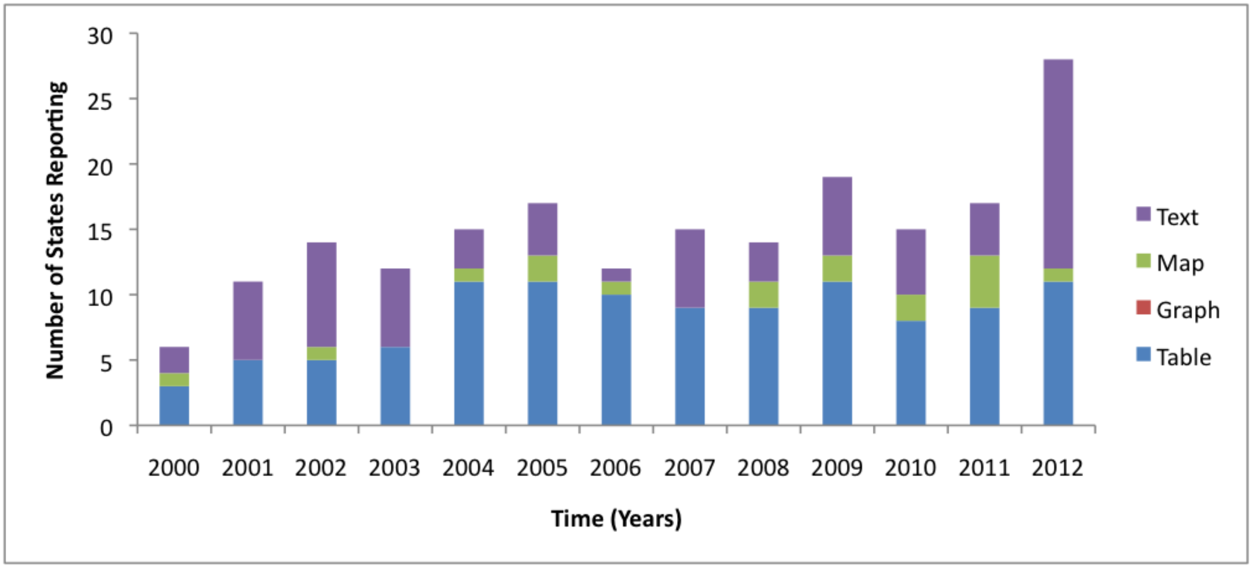

Figure S11: Agriculture agency presentation of data by year.

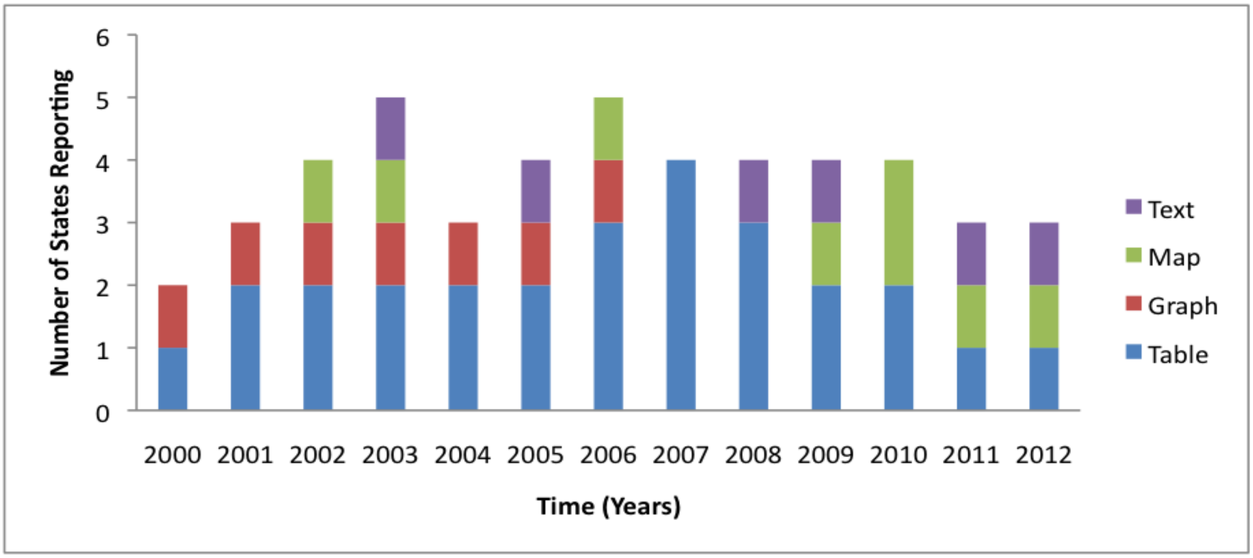

Figure S12: Wildlife agency presentation of data by year.

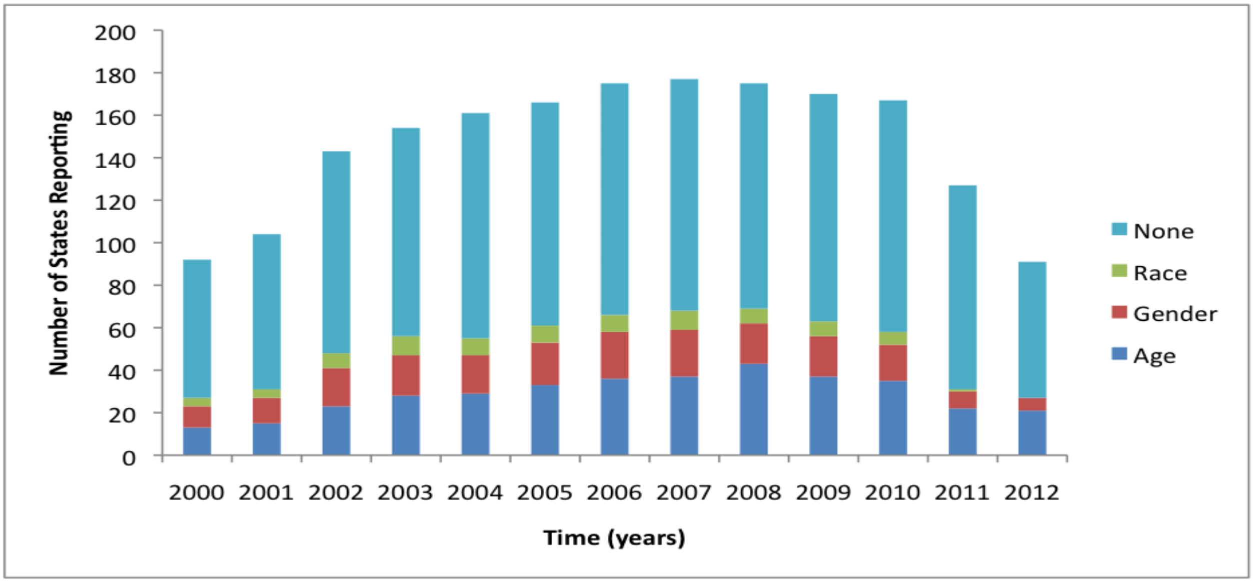

Figure S13: Public health agency demographics of data by year.

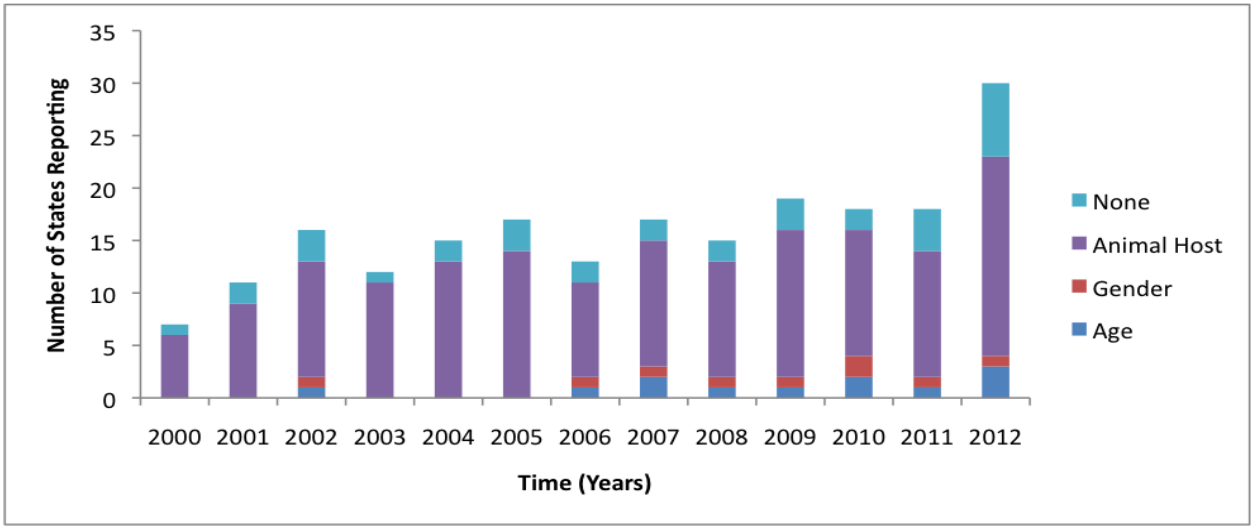

Figure S14: Agriculture agency demographics of data by year.

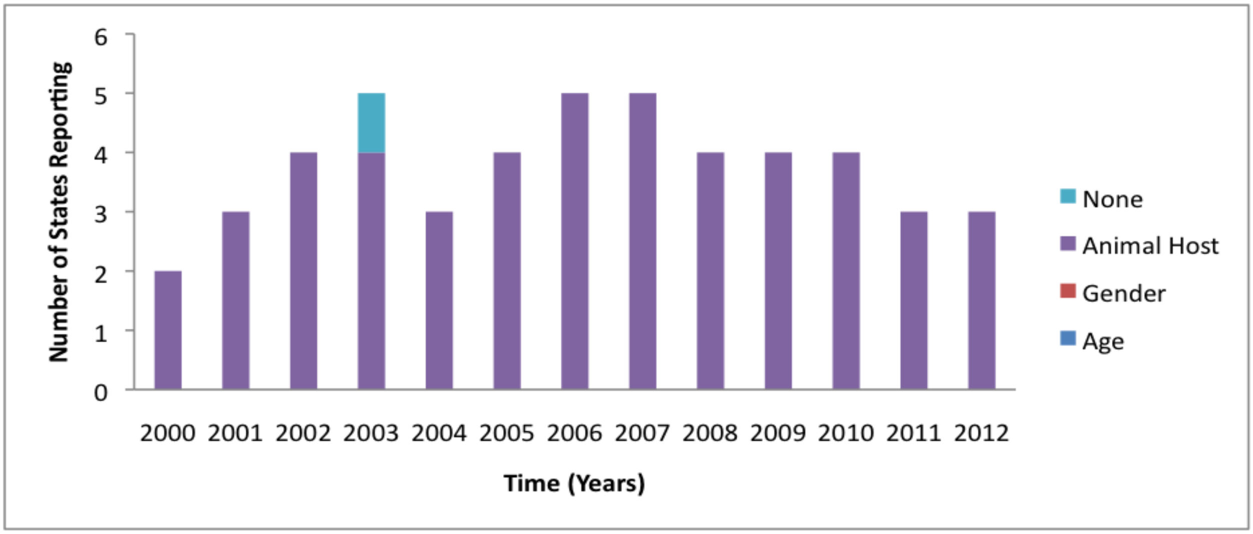

Figure S15: Wildlife agency demographics of data by year.

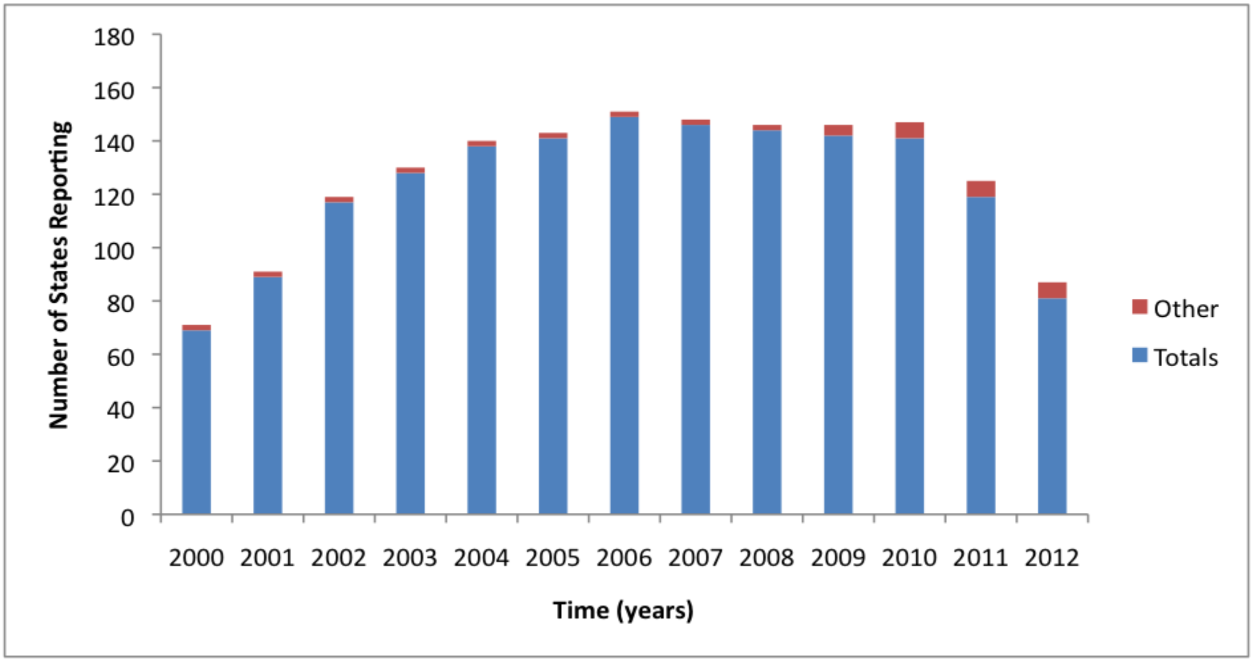

Figure S16: Public health agency statistics of data by year.

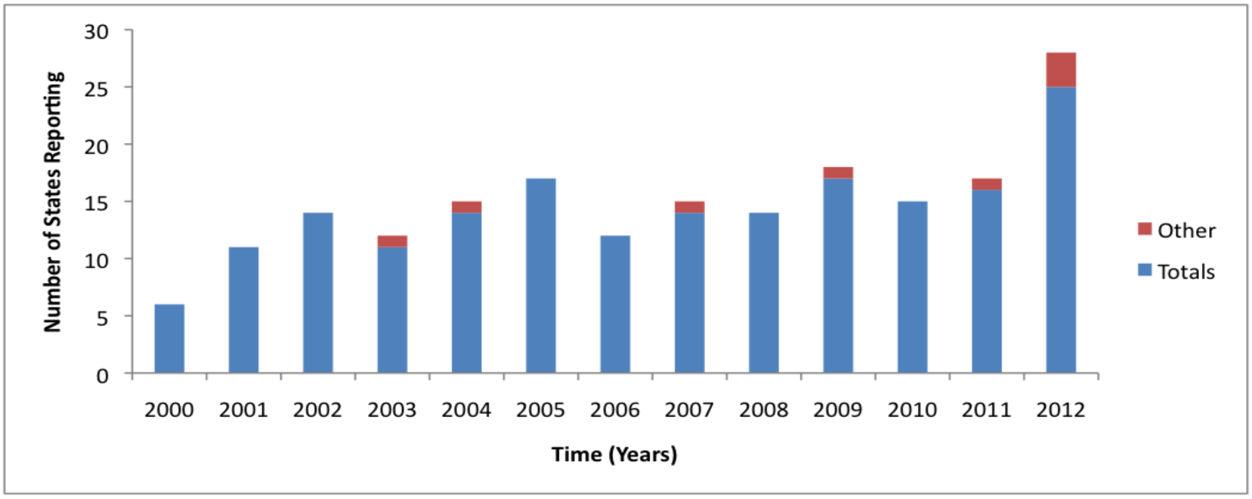

Figure S 17: Agriculture agency statistics of data by year.

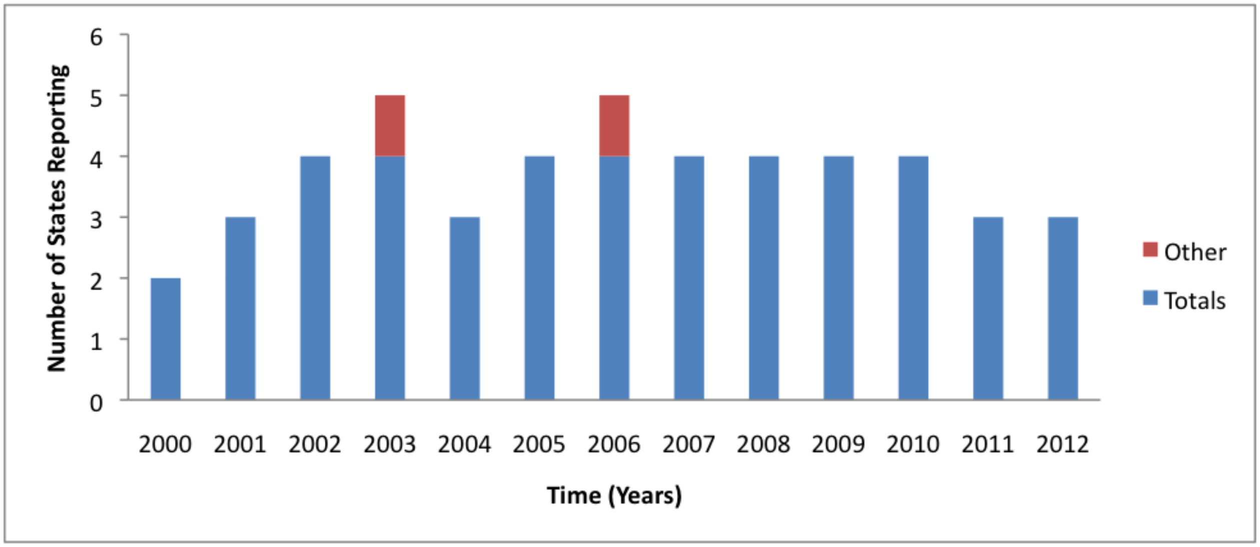

Figure S18: Wildlife agency statistics of data by year.
